# Peroxidase-Like Catalytic Activity of Ag_3_PO_4_ Nanocrystals Prepared by a Colloidal Route

**DOI:** 10.1371/journal.pone.0109158

**Published:** 2014-10-01

**Authors:** Yuanjun Liu, Guoxing Zhu, Jing Yang, Aihua Yuan, Xiaoping Shen

**Affiliations:** 1 School of Environmental and Chemical Engineering, Jiangsu University of Science and Technology, Zhenjiang, China; 2 School of Chemistry and Chemical Engineering, Jiangsu University, Zhenjiang, China; 3 State Key Laboratory of Coordination Chemistry, Nanjing University, Nanjing, P. R. China; University of Akron, United States of America

## Abstract

Nearly monodispersed Ag_3_PO_4_ nanocrystals with size of 10 nm were prepared through a colloidal chemical route. It was proven that the synthesized Ag_3_PO_4_ nanoparticles have intrinsic peroxidase-like catalytic activity. They can quickly catalyze oxidation of the peroxidase substrate 3, 3, 5, 5-tetramethylbenzidine (TMB) in the presence of H_2_O_2_, producing a blue color. The catalysis reaction follows Michaelis-Menten kinetics. The calculated kinetic parameters indicate a high catalytic activity and the strong affinity of Ag_3_PO_4_ nanocrystals to the substrate (TMB). These results suggest the potential applications of Ag_3_PO_4_ nanocrystals in fields such as biotechnology, environmental chemistry, and medicine.

## Introduction

Owing to its excellent photocatalytic properties and broad range of applications such as in water-splitting, photocatalytic reactions, silver phosphate has got extensive study and has become a well studied material [1]. Especially, partly owing to the highly dispersive Ag *s*-Ag *s* bands without localized *d* states [2], Ag_3_PO_4_ semiconductor exhibits extremely high photooxidative ability for O_2_ evolution from water as well as organic dye decomposition under visible light irradiation [3]–[6]. A much higher quantum efficiency (up to 90%) than the previously reported values at wavelengths longer than 420 nm was also achieved with it [1].

Up to now, various methods have been proposed to further enhance and optimize the photoelectric and photocatalytic properties of Ag_3_PO_4_ *via* microstructure control or forming composites with other components to improve its stability, bandgap structure and surface area [7]–[12]. Although extensive studies have been made for the photocatalytic applications of various Ag_3_PO_4_ micro-/nanoparticles and their composites, the application of Ag_3_PO_4_ in biological systems, for example used as biocatalyst, has rarely been studied, while the presence of phosphorus in biological systems is well known.

Recently, it was found that Fe_3_O_4_ nanoparticles have intrinsic enzyme-like activity similar to peroxidases found in nature, though Fe_3_O_4_ are usually thought to be biological and chemical inert [13]. After that, several kinds of micro/nanoparticles with smaller size or special structure were prepared for developing enzyme mimics, including the ferromagnetic nanoparticles with peroxidase-like activity [14]–[23], ceria oxide nanoparticles [24]–[27], and V_2_O_5_ nanowires [28], carbon-based nanomaterials [29]–[36] and so on [37]–[42]. In contrast to natural enzymes, nanoparticles-based enzyme mimics own prominent advantages. First, they have greater resistance to extremes of pH and temperature, while natural enzymes are usually sensitive to the external conditions and also easily lose their activity. Secondly, nanoparticles-based mimic enzymes have higher stability, while natural enzymes can be digested by proteases. Thirdly, with the extensive development of nanoscience and nanotechnology in the past three decades, the preparation and surface modification of various nanoobjects can be easily carried out, while the synthesis and purification of natural enzymes are still time-consuming, expensive, and also difficult [14].

Exploitation of new functions of known nanomaterials is one of the most attractive aspects in nanoscience [37]. Inspired by the above pioneering research, we investigated the peroxidase-like activity of Ag_3_PO_4_ nanocrystals, considering that some Ag-based metal alloy nanoparticles own intrinsic peroxidase-like activity. Ag_3_PO_4_ nanoparticles with smaller size were obtained via a simple colloidal route. It was found that the obtained Ag_3_PO_4_ nanoparticles show their ability to catalyze peroxidatic reactions in aqueous media. The kinetic parameters were also tested and compared. The reaction catalyzed by these Ag_3_PO_4_ nanoparticles followed a Michaelis-Menten kinetic behavior with an excellent catalytic activity, making it a promising mimic of peroxidase. The new application of Ag_3_PO_4_ as peroxidase mimic will add new content to this interesting material.

## Results and Discussion

**Figure 1:**
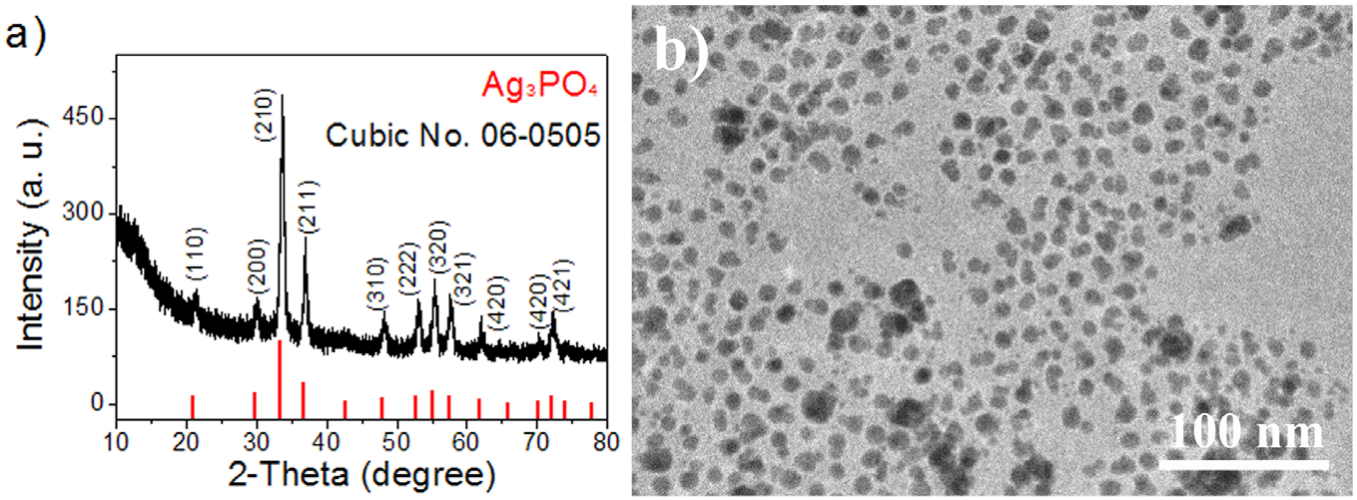
The prepared Ag_3_PO_4_ nanoparticles. a) XRD pattern and b) TEM image, the standard pattern of cubic Ag_3_PO_4_ with JCPDS No. 06-0505 is also shown for comparison.

A colloidal route was employed for the preparation of Ag_3_PO_4_ nanoparticles because it can produce Ag_3_PO_4_ nanoparticles with smaller size [6]. The preparation was carried out at room temperature with H_3_PO_4_ and AgNO_3_ as raw materials, while toluene and oleylamine were used as solvent and surfactant. The crystal phase of the obtained Ag_3_PO_4_ nanoparticles was first determined by X-ray diffraction (XRD). Fig. 1a shows the corresponding XRD pattern, which can be easily indexed to cubic Ag_3_PO_4_ with JCPDS No. 06-0505. The relatively strong peaks at 21.3, 30.1, 33.6, 36.9, 53.1, 55.3, and 57.6^o^ corresponds to the (110), (200), (210), (211), (222), (320), and (321) crystal planes of cubic Ag_3_PO_4_, respectively. No diffraction peak from Ag with zero-valent state is observed in the pattern. This reveals pure Ag_3_PO_4_ is obtained with this simple route. It should be noted that the XRD pattern shows relatively broad peak, indicating the smaller size of Ag_3_PO_4_ nanocrystals according to Scherrer formula. Fig. 1b shows a typical transmission electron microscopy (TEM) image of the obtained Ag_3_PO_4_ nanocrystals, from which spherical particles with small size are observed. The Ag_3_PO_4_ particles show relatively uniform size. The average diameter is about 10 nm.

**Figure 2:**
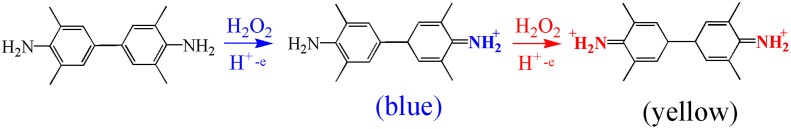
The oxidation reaction of TMB.

Before property investigation, the obtained Ag_3_PO_4_ nanocrystals were firstly treated through usual ligand exchange route to transfer it to being hydrophilic state. Peroxidase-like activity of the Ag_3_PO_4_ nanocrystals was evaluated in the catalysis oxidation of a commonly used peroxidase substrate, 3, 3′, 5, 5′-tetramethylbenzidine sulfate (TMB), in the presence of H_2_O_2_. TMB is colorless and can be oxidized slowly by H_2_O_2_ (Fig. 2). The often observed oxidation products are two colored products [43]. The first product is a blue charge-transfer complex of diamine, which are formed in rapid equilibrium with the radical cation. Its maximal absorption wavelength locates at ∼370 and ∼652 nm. Another product is a yellow diimine, which is generated by further oxidation of the diamine with excess H_2_O_2_ or strong acidic condition. The diimine product is stable in acidic conditions with maximal absorption wavelength of 450 nm. The first-step reaction with the formation of blue diamine is often used as a model process to evaluate activity of peroxidases.

**Figure 3:**
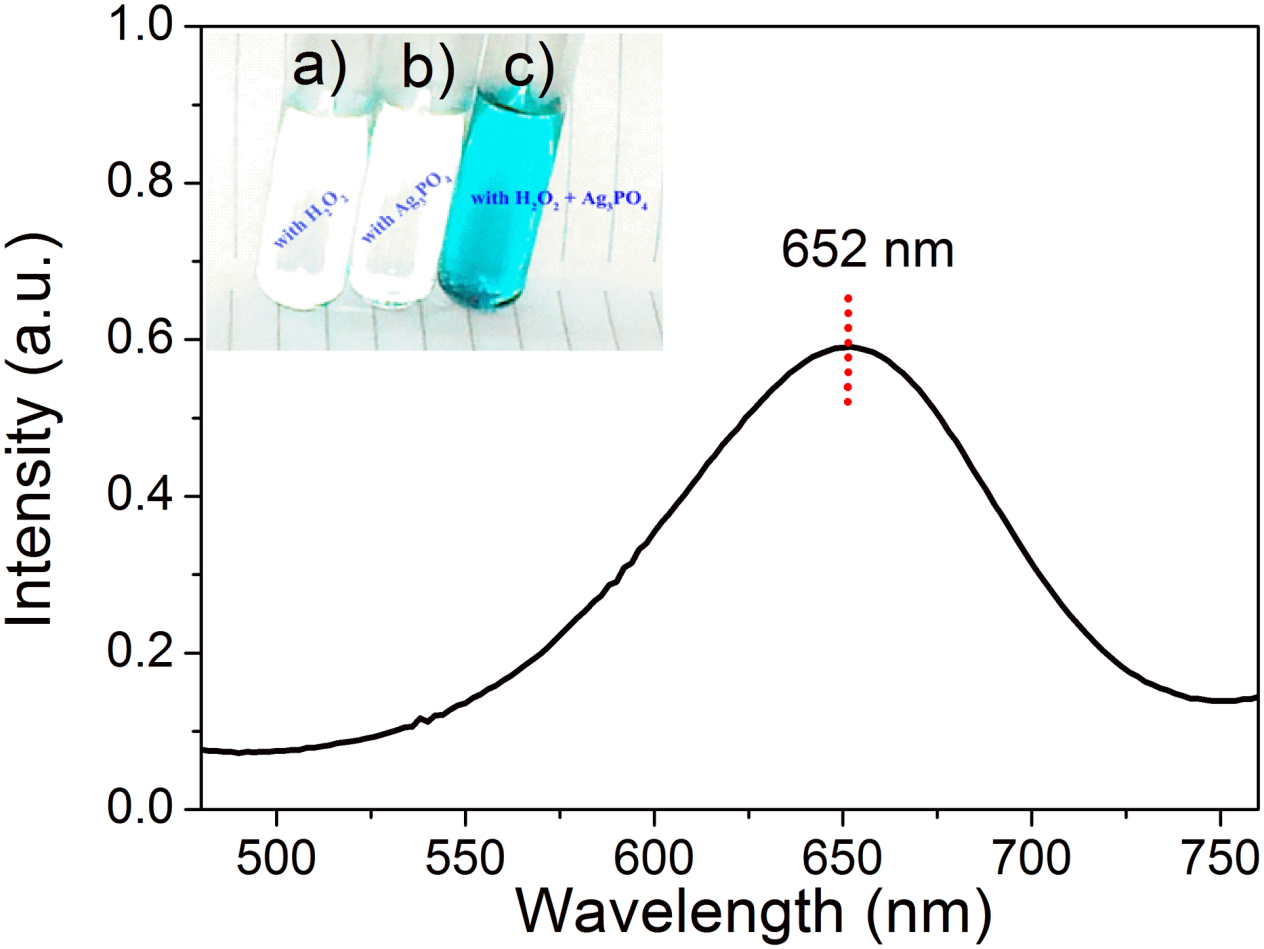
UV-vis spectrum of the reaction system with H_2_O_2_ + Ag_3_PO_4_ nanocrystals. The inset shows images of oxidation color reaction of TMB in NaAc buffer with a) H_2_O_2_, b) Ag_3_PO_4_ nanocrystals and c) H_2_O_2_ + Ag_3_PO_4_ nanocrystals. Reaction conditions: 0.3 mM of TMB, 2 mg/mL of Ag_3_PO_4_ (if with), 3.6 mM of H_2_O_2_ (if with) in 5 mL of NaAc buffer with pH 4.0. The reaction proceeded at 25°C with time of 30 min.

As can be seen in Figure 3, our preliminary experiment shows that Ag_3_PO_4_ nanocrystals can catalyze the oxidation of TMB by H_2_O_2_ in NaAc buffer producing a blue solution (inset of Figure 3), suggesting the formation of charge-transfer complex of diamine. The typical absorbance peak of this oxidation product of TMB is at 652 nm. The reaction system will turn to be yellow if it was overnight placed, which is due to the formation of diimine. Also, it was found that Ag_3_PO_4_ nanocrystals or H_2_O_2_ alone did not produce significant color change (inset of Figure 3). These results confirm that Ag_3_PO_4_ nanocrystals behave with peroxidase-like activity toward TMB.

**Figure 4:**
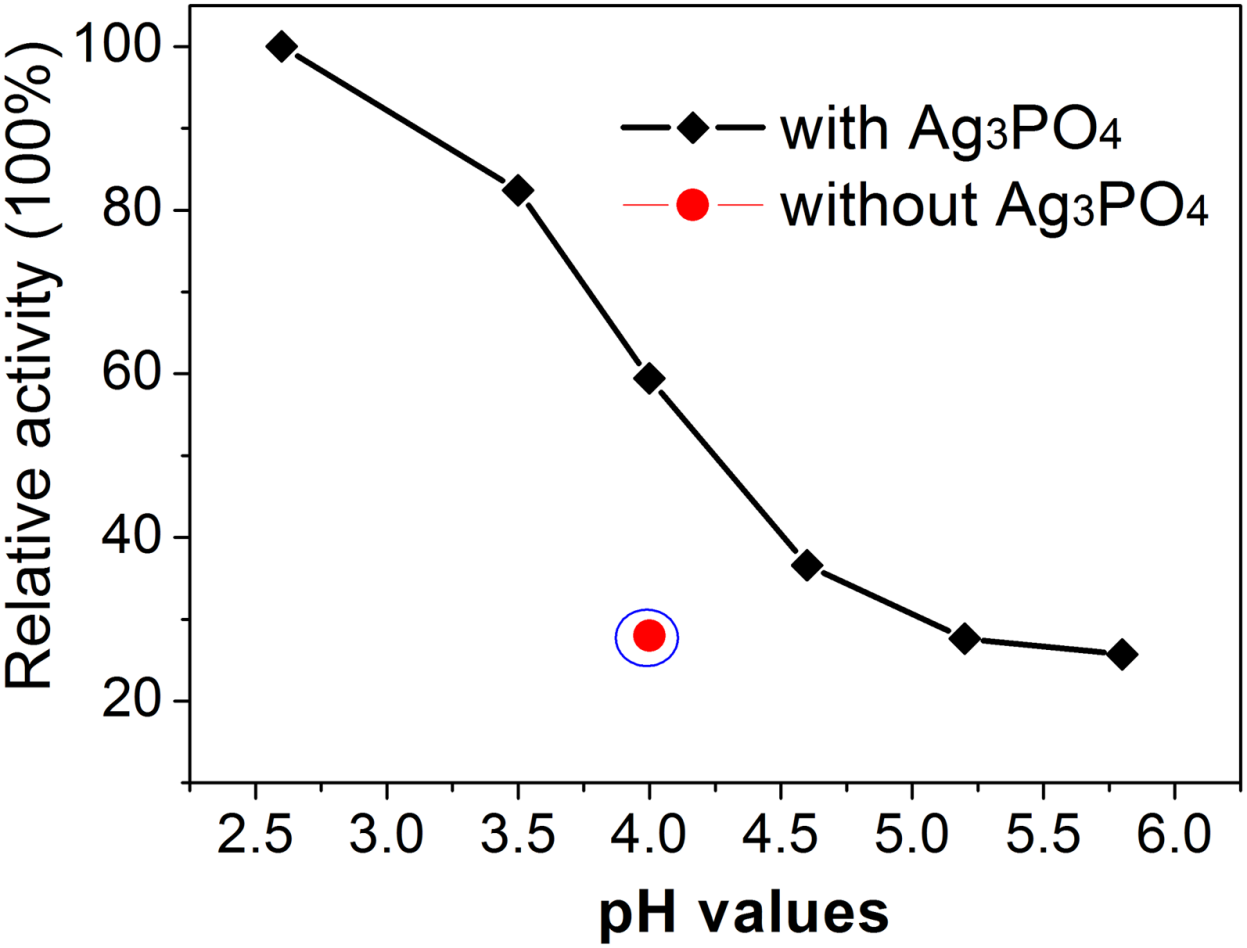
pH value-dependent peroxidase-like catalytic activity of Ag_3_PO_4_ nanocrystals. Reaction conditions: 0.3 mM of TMB, 2 mg/mL of Ag_3_PO_4_, 3.6 mM of H_2_O_2_ in 5 mL of NaAc buffer with different pH values. The reaction proceeded at 25°C with time of 30 min.

To investigate the effect of pH values of buffer solution on catalytic properties, we performed the catalytic experiments in NaAc buffer with different pH values. Relative activity was analyzed based on the absorption at 652 nm. Fig. 4 shows the relative activity of the Ag_3_PO_4_ nanocrystals with reaction time of 30 min at room temperature. It was found that the catalytic activity of Ag_3_PO_4_ nanocrystals is significantly affected by pH values. Only very lower catalytic activity was demonstrated when the pH value of buffer exceeds 4.5. We then selected the buffer with pH of 4 for the subsequent study due to the consideration of the possible disability of Ag_3_PO_4_ nanocrystals in buffer with strong acidity. With the buffer of pH = 4, a contrast measure was conducted in the absence of Ag_3_PO_4_ nanocrystals, which give very low absorbency at 652 nm.

**Figure 5:**
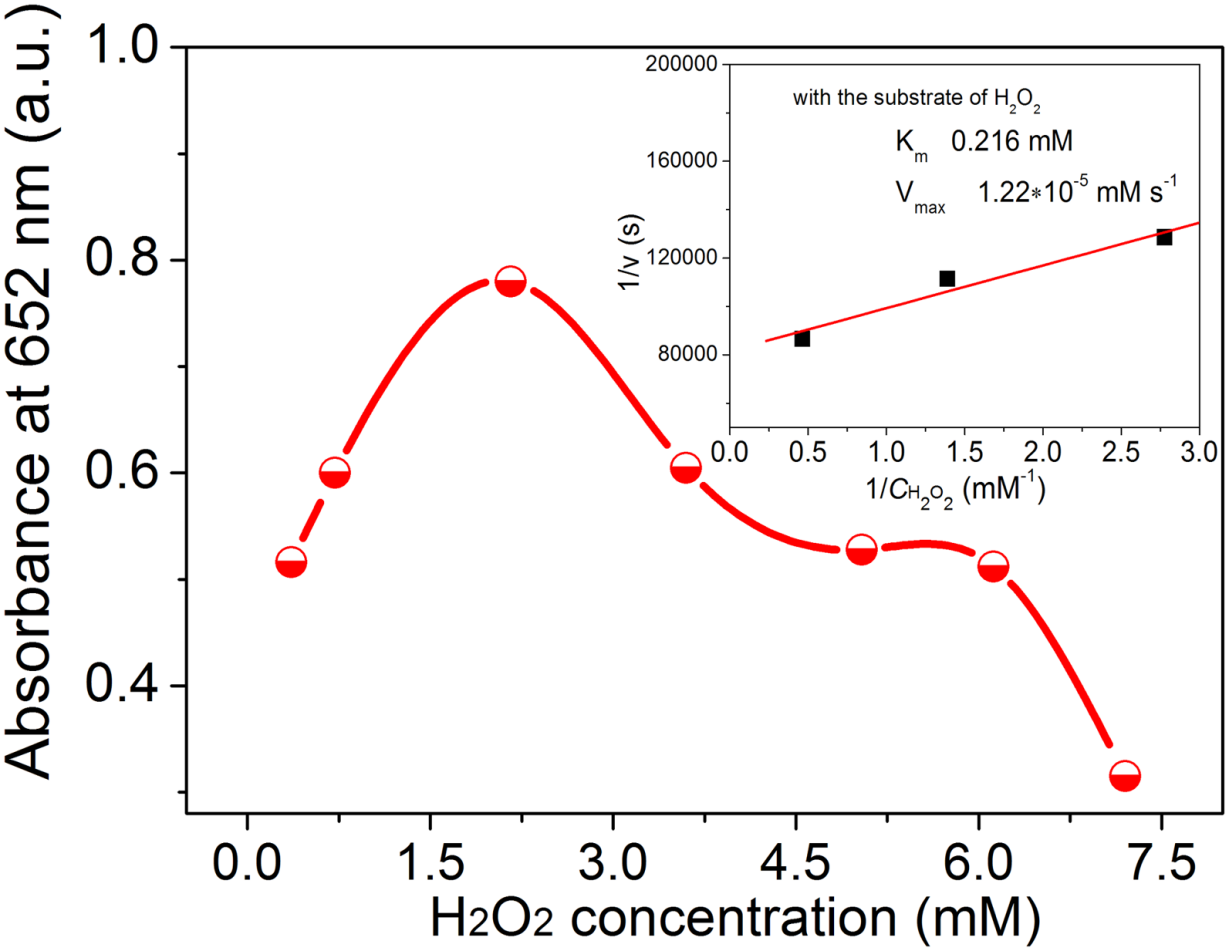
H_2_O_2_ concentration dependent peroxidase-like catalytic activity of Ag_3_PO_4_ nanocrystals. Reaction conditions: 0.3 mM of TMB, 2 mg/mL of Ag_3_PO_4_ in 5 mL of NaAc buffer with pH 4.0. The concentration of H_2_O_2_ varies in the range of 0.36–7.2 mM. The reaction proceeded at 25°C with time of 30 min.

As shown in Fig. 5, the catalytic activity of Ag_3_PO_4_ nanocrystals is also H_2_O_2_ concentration dependent. With the increasing of H_2_O_2_ concentration, the peroxidase-like catalytic activity increases at first. When the concentration of H_2_O_2_ reaches about 2.2 mmol/L (that is 7.3 times that of TMB), the catalytic activity of the Ag_3_PO_4_ nanocrystals achieves its highest point. However, further increasing the H_2_O_2_ concentration causes a lower absorbance at 652 nm, which implies low catalytic activity at higher H_2_O_2_ concentration. In fact, many nanoparticle-based enzyme mimics show this kind hump-shaped relationship between H_2_O_2_ concentration and the reaction activity including the highly studied enzyme mimic material Fe_3_O_4_ [13], [44]–[46]. This phenomenon is also similar to that observed with horseradish peroxidase [13], [44]. It is reasonable that the reaction activity increases at first with the increase of H_2_O_2_ concentration, since more oxidant is involved in the reaction system. With high concentration of H_2_O_2_ in the reaction system, it is usually proposed that the H_2_O_2_ moleculars would cap on the surface of catalyst, inhibiting the attachment of substance to the surface of catalyst, and so weakening the catalytic activity. Thus, a hump-shaped relationship is obtained. While, this H_2_O_2_ concentration dependent catalytic activity is different from that of CuO [47], Au nanoparticles [38], Ag nanoparticles [48]. In those cases, the reaction actitvity increases monotonously with H_2_O_2_ concentration till a saturation state is obtained.

For biomolecular enzymes, the catalytic active center is usually the coordination unsaturated metal sites under the capping of protein networks. For nanoparticles, the surface atoms place in similar situation–coordination unsaturation under the capping of surfactant moleculars. Thus, it is possible that they may share some common points in catalytic process, although the catalysis mechanism of inorganic catalysts and enzymes are usually different. At present stage, the Michaelis-Menten model is widely used for the study of nanoparticle-based enzyme mimetics [13], [26], [49]–[52]. Therefore, in our study, the Michaelis-Menten model was also selected to understand the peroxidase-like catalytic activity of the Ag_3_PO_4_ nanocrystals.

**Figure 6:**
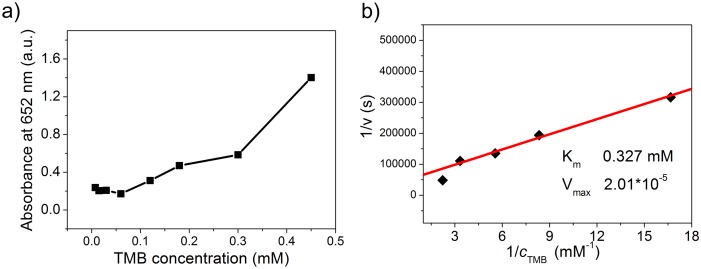
Steady-state kinetic assay of the Ag_3_PO_4_ nanocrystals. a) The concentration of H_2_O_2_ was 5 mM and the TMB concentration was varied. b) Double reciprocal plots of *1/V ∼1/C_TMB_*. Reaction conditions: 2 mg/mL of Ag_3_PO_4_ in 5 mL of NaAc buffer with pH 4.0 at 25°C.

For further analyzing the catalytic kinetic parameters, the catalytic activity of Ag_3_PO_4_ nanocrystals was studied by the Michaelis-Menten model with TMB as substrate. The apparent steady-state kinetic parameters for the reaction were determined at 25°C with pH = 4 buffer. Fig. 6a shows the TMB concentration dependent catalytic activity. The absorbance increases with the increasing of TMB concentration, especially at higher TMB concentration range. Absorbance data were then back-calculated to concentrations by the Beer-Lambert Law using a molar absorption coefficient of 39000 M^−1 ^cm^−1^ for TMB-derived oxidation product [53].

Kinetic parameters were then calculated based on the Michaelis–Menten equation, which
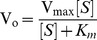
(1)describes the relationship between the rate of substrate conversion by an enzyme catalyst and the concentration of the substrate. In this equation, *V_o_* is the conversion rate, *V_max_* is the maximum conversion rate, *[S]* is the substrate concentration, and *K_m_* is the Michaelis constant which denotes the affinity of the enzyme. According to (1), a corresponding plot of *1/V_o_∼1/[S]* is shown in Fig. 6b, which reveals that the reaction catalyzed by Ag_3_PO_4_ nanocrystals follows Michaelis-Menten kinetics in a certain range of substrate concentrations.

The corresponding values of K_m_ and V_max_ calculated from double reciprocal plots with TMB substrate are 0.327 mM and 2.01×10^−5 ^mM s^−1^ (Fig. 6b). By comparing the apparent kinetic parameters, the K_m_ value of Ag_3_PO_4_ nanocrystals with TMB substrate is lower than that of reported horserahish peroxidase (HRP, 0.434) tested in buffer solution with pH 3.5 at 40°C [13], suggesting that Ag_3_PO_4_ nanocrystals have a higher affinity for TMB than HRP. The V_max_ value is similar to that of typical nanomaterial-based enzyme mimetics, Fe_3_O_4_ nanoparticles. The values of K_m_ and V_max_ calculated with H_2_O_2_ substrate are 0.216 mM and 1.27×10^−5 ^mM s^−1^ (inset of Fig. 5). The K_m_ value obtained here with H_2_O_2_ substrate is lower than that of Fe_3_O_4_ nanoparticles and HRP [13], [19] reported under similar test conditions (buffer solution with pH 3.6–4.6, TMB concentration of 0.2–0.8 mM, temperature of 25°C), suggesting that the Ag_3_PO_4_ nanocrystals exhibit strong affinity towards TMB and H_2_O_2_. The strong affinity would be a reason for the excellent peroxidase-like activity.

**Figure 7:**
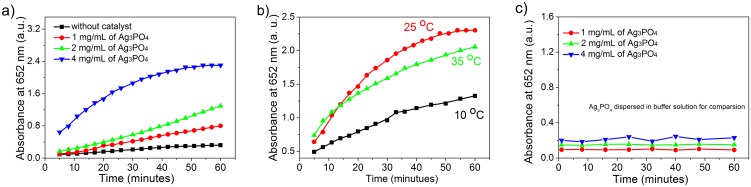
Time-dependent catalytic activity with a) different amounts of Ag_3_PO_4_ nanocrystals at 25°C and b) at different temperature with 4 mg/mL of Ag_3_PO_4_ nanocrystals. Reaction conditions: 0.3 mM of TMB, 0–4 mg/mL of Ag_3_PO_4_, 3.6 mM of H_2_O_2_ in 5 mL of NaAc buffer with pH of 4. c) Control experiment to show the time-dependent absorption spectra of Ag_3_PO_4_ nanocrystals dispersed in buffer solution (pH = 4) at different concentrations (1, 2, 4 mg/mL).

Fig. 7 shows the time-dependent catalytic activity of four similar reaction systems with different amounts of Ag_3_PO_4_ nanocrystals. The absorbance of the Ag_3_PO_4_ catalyzed system is much higher than the one without Ag_3_PO_4_ catalyst. Among the three Ag_3_PO_4_ catalyzed systems, the more catalyst is involved, the higher absorbency is shown. Control experiment to examine the time-dependent absorption spectrum of Ag_3_PO_4_ nanocrystals dispersed in buffer solution (pH = 4) at different concentrations (1, 2, 4 mg/mL) gives no obvious change of absorbency with time, indicating that the increasing absorbency in reaction system is related to the oxidation of TMB, but not originated from the increased concentration of Ag_3_PO_4_ nanocrystals (Fig. 7c). These results show that higher reaction rates are obtained with high concentration of Ag_3_PO_4_ catalyst. With 4 mg/mL of Ag_3_PO_4_ nanocrystals, it seems that the reaction arrives equilibrium at about 50 minutes. The influence of temperature (10°C, 25°C, 35°C) on the peroxidase-like catalytic activity is also investigated with 20 mg of Ag_3_PO_4_ nanocrystals. As shown in Fig. 7b, the Ag_3_PO_4_ nanocrystals show the higher peroxidase-like catalytic activity at temperature of 25°C, although in the initial 15 min, the system has a relatively higher reaction rate at 35°C. The temperature dependent catalytic activity is similar to that of natural enzyme or Fe_3_O_4_ nanoparticles, which have a preferred temperature.

The use of Ag_3_PO_4_ nanocrystals as catalysts for electron-transfer reactions has been rarely investigated, and the in-depth catalytic mechanism is also not clear at present stage. It is proposed Ag_3_PO_4_ nanocrystals plays a role of transferring electrons to hydrogen peroxide, causing them to decompose. It is assumed that the oxygen-oxygen bond of H_2_O_2_ will rapidly broken by the catalytic action of Ag_3_PO_4_ nanocrystals to give OH radicals. The OH radicals stabilize at the surface of the Ag_3_PO_4_ nanocrystals, and react with TMB.

In summary, colloidal Ag_3_PO_4_ nanocrystals with smaller size were prepared. The peroxides-like catalytic activity of these Ag_3_PO_4_ nanocrystals were systematically investigated. The results show that they have a higher activity at acid environment. The catalytic activity was also dependent on H_2_O_2_ concentration, temperature, and catalsy amount. Kinetic analysis indicates that the catalysis reaction is in accord with typical Michaelis-Menten kinetics. The apparent kinetic parameters suggest the higher affinity of Ag_3_PO_4_ nanocrystals than that of horserahish peroxidase. Our research gives new content to well-known Ag_3_PO_4_ material and provides a new nanomaterial-based peroxide enzyme mimitics, which would found applications in medical diagnostics and biochemistry.

## Materials and Methods

Synthesis of Ag_3_PO_4_ nanocrystals: Ag_3_PO_4_ nanocrystals were synthesized with reported methods with minor adjustments [6]. In brief, 8.5 g of AgNO_3_ and 32 mL of oleylamine were dispersed in 150 mL of toluene and stirred for about 2 h at room temperature. After AgNO_3_ was fully dissolved, an ethanol solution containing 50 mL of ethanol, 2 mL of H_2_O, 2.84 mL of H_3_PO_4_ was added into the above solution. The solution tuned into yellow colloid quickly. After reaction for 30 mins at room temperature, Ag_3_PO_4_ nanocrystals were precipitated by adding ethanol, and washed several times with toluene and ethanol. The dark-yellow precipitate was dried in an oven.

Characterization of Ag_3_PO_4_ nanocrystals: The phase structure of the as-synthesized products were characterized using X-ray diffraction (XRD, Bruker D8 ADVANCE) with Cu-Kα radiation (λ = 1.5406 Å) at a scanning rate of 6° min^−1^. The morphology and size of the products were examined by a transmission electron microscope (TEM, JEOL JEM-2100) with an accelerating voltage of 200 kV. The Ag_3_PO_4_ product dispersed in ethanol was dropped onto a holey copper grid covered with an amorphous carbon film for the TEM examination.

Surface modification for Ag_3_PO_4_ nanocrystals: Before property investigation, the obtained Ag_3_PO_4_ nanocrystals were firstly treated through usual ligand exchange route [54] to transfer it to being hydrophilic state. Briefly, about 50 mg of Ag_3_PO_4_ nanocrystals were dispersed into the mixture of hexane (35 mL), distilled water (15 mL), and ethanol (30 mL) through magnetic stirring. Then, 6-amino caproic acid (0.13 g) and equivalent molar NH_3_·H_2_O in 5 mL of distilled water was added into the above system. After that, the mixture was heated to 70°C and kept at that temperature for 4 h. The nanocrystals were then collected by centrifugation and washed with water. Through this process, the hydrophobic Ag_3_PO_4_ nanocrystals were transformed into hydrophilic state, which can be dispersed in water.

Peroxidase-like catalytic activity of Ag_3_PO_4_ nanocrystals: The peroxidase-like activity of freshly treated Ag_3_PO_4_ nanocrystals was determined by measuring the formation of a blue charge-transfer complex of diamine from TMB at 652 nm (ε = 39000 M^−1 ^cm^−1^). The TMB oxidation activity measurement, unless otherwise specified, was conducted in sodium acetate buffer (pH 4.0) in the presence of Ag_3_PO_4_ nanocrystals (2 mg mL^−1^) with 0.3 mM of TMB and 3.6 mM of H_2_O_2_. The reaction proceeded at 25°C with time of 30 min.

pH Measurements: The activity of the Ag_3_PO_4_ nanocrystals at different pH values was performed using the same conditions as above, except different buffer compositions (with different concentration ratios of HAc to NaAc) for different pH values were employed. The reaction was carried out with 2 mg mL^−1^ of Ag_3_PO_4_ nanocrystals to which TMB (0.3 mM) and H_2_O_2_ (3.6 mM) were added. The pH of the different buffers was adjusted by using a pH meter.

Determination of kinetic parameters: The steady-state kinetics were performed by varying one of the concentrations of Ag_3_PO_4_ nanocrystals (0–4 mg mL^−1^), H_2_O_2_ (0.35–7 mM), or TMB (0–0.45 mM) at a time. The reaction was carried out in acetate buffer (pH 4.0) for 30 min and monitored by measuring the absorbency at 652 nm. The kinetic curves were adjusted according to the Michaelis-Menten model.
